# Untapped potential of non-conventional *rubus* species: bioactivity, nutrition, and livelihood opportunities

**DOI:** 10.1186/s13007-023-01094-y

**Published:** 2023-10-27

**Authors:** Saurav Chandra Bhatt, Bindu Naik, Vijay Kumar, Arun Kumar Gupta, Sanjay Kumar, Manpreet Singh Preet, Nitya Sharma, Sarvesh Rustagi

**Affiliations:** 1 Department of Food Science and Technology, Graphic Era (Deemed to Be University), Bell Road, Clement Town, Dehradun, 248002 Uttarakhand India; 2 Himalayan School of Biosciences, Swami Rama Himalayan University, Jolly Grant, Dehradun, 248016 Uttarakhand India; 3 School of Agriculture, Graphic Era Hill University, Clement Town, Dehradun, Uttarakhand India; 4 World Resources Institute India, Hauz Khas, New Delhi, 110016 India; 5 Department of Food Technology, UCALS, Uttaranchal University, Dehradun, Uttarakhand India

**Keywords:** *Rubus*, Superfood, Nutraceutical, Phytochemicals, Anticancer, Antimicrobial

## Abstract

*Rubus* species holds promise as a valuable source of polyphenols and bioactive compounds, offering significant potential as functional food ingredients with both nutraceutical and pharmaceutical benefits. However, many edible species within this genus remain under-explored and their importance is largely unrecognized. This review aims to provide an overview of the nutritional and bioactive components of both explored and under-explored *Rubus* species, highlighting their potential health advantages, value addition, and recent advancements. The economic exploitation of *Rubus* is currently limited to a few cultivated species, while numerous non-conventional and wild edible species are overlooked. Recognizing the economic and nutritional significance of exploited *Rubus* species, it is imperative to explore the untapped potential of these underutilized plants. By doing so, these species can be preserved from endangerment and contribute to nutritional and livelihood security for communities having access to them. This review emphasizes the importance of understanding the exceptional characteristics of *Rubus* species as "superfoods" and encourages the promotion and cultivation of these unexplored species. By expanding the cultivation and utilization of under-explored *Rubus* species, we can unlock their full potential and support sustainable nutritional and economic benefits.

## Introduction

The *Rubus* genus is part of the Plantae kingdom and Rosaceae family. It is widely distributed across the world and exists at 4500 m from sea level [1]. It is a diverse group of plants that includes a variety of species such as Himalayan berries (*Rubus ellipticus*), blackberries (*Rubus fruticosus*), raspberries (*Rubus ideaus*), dewberries (*Rubus flagellaris*), and so on. *Rubus* is a genus comprising 12 subgenera that also covers a diverse range of wild species [2]. While the genus comprises more than 700 species but among them, only a few are commercialized including black raspberry (*Rubus occidentalis*), red raspberry (*Rubus ideaus* L.), raspberry (*Rubus chingii* Hu), and blackberry (*Rubus fruticosus* L.) [3]. Most of the plants in the genus such as *R. ellipticus, R. nives, and R. ulmifolius* contain woody stems with prickles like roses. Many of the species in this genus, including raspberry, black raspberry, and Himalayan raspberry are widely consumed as fruits and they are valued for their sweet, juicy fruits that are high in vitamins, minerals, and antioxidants. Fruits are commonly used for a variety of purposes, including fresh consumption, juice production, and the preparation of jams, jellies, and syrups. To date, there is a lack of practical evidence to support the notion that species within the *Rubus* genus possess toxic properties. Most of these plants have common characteristics like spines, bristles, and gland-tipped hairs. The phenomenon of hermaphroditism, characterized by the presence of both male and female reproductive organs, is seen in the most of species within the *Rubus* genus, except *Rubus chamaemorus* [1]. These species have gained popularity for their industrial exploitation, while some species hold significant value but are not fully utilized. Even at present, some researcher has mentioned *R.ideaus* and *R.fruticosus* as wild variety [4, 5], however, the proper research and value addition of these varieties made them a good source of nutraceutical and pharmaceutical products. Similarly, it is important to conduct similar work for the other non-conventional plants within the genus to raise awareness about the benefits these underexplored varieties provide.

Non-conventional edible plants are categorized as the plants which are non-popular in the human diet or animal fodder, and they can be called “plants of the future”, “alternative food plants”, “Famine foods”, “Wild edible plants”, “traditional vegetables” [6] as they are said to have nutritious and medicinal values that play an important function in the human diet [7]. The use of these plants is said to be very specific to an individual community or being neglected [6]. The modern livelihood lacks knowledge about the importance of these fruits and the circulation of awareness, using factors such as field trips, potential research, or social media, to tell people about the potential health benefits of non-conventional plants may be an interesting strategy to promote human nutritional variations. Wild fruits and semi-wild fruits can be considered into the category of non-conventional edible plants. These plants grow naturally in forests, and discarded lands. The non-conventional (wild) *Rubus* species include species such as *Rubus rosifolius* (west Indian Raspberry) [7], *Rubus liebmanni* [8], *Rubus palmeri* [9], *Rubus ellipticus* (Himalayan raspberry) [10], *Rubus macilentus* (Lean Raspberry) [11], etc. In India, *Rubus assamensis* (Assam Raspberry)*, Rubus biflorus* (Two-Flower raspberry)*, Rubus calycinoides* (Darjeeling Raspberry)*, Rubus ellipticus* (yellow Himalayan Raspberry)*, Rubus indicus* (Indian Raspberry)*, Rubus macilentus* (Lean Raspberry)*, Rubus moluccanus* (wild Raspberry)*, Rubus nepalensis* (Nepalese Raspberry)*, Rubus niveus* (Mysore Raspberry)*, Rubus paniculatus* (Heart-leaf Raspberry)*, Rubus pedunculosus* (Three leaf raspberry)*, Rubus rosifolius* (Roseleaf bramble)*, Rubus Rosifolius* var*. coronarius* (Roseleaf Raspberry)*, and Rubus ulmifolius* (Elm-Leaf Raspberry) are some of the commonly found species (https://www.flowersofindia.net/) but commercialization of these are not popular in the industry or individual communities. To the best of our knowledge, no review has yet presented the bioactive components and their applications, value addition, and recent developments of *Rubus* species. As such the current review intends to describe the chemical and bioactive components of both explored and under-explored fruits and other plant parts of the *Rubus* species, including their potential health benefits the related mechanism of action of compounds, and recent advancements. The current work is designed to promote the cultivation of underutilized species of this genus as some of the plants of the same genus are well explored for their nutraceutical properties and their value-added products provide livelihood security. Additionally, it provides a comparison of the *Rubus* genus's cultivated species with some of its less-studied non-cultivated species, highlighting their commercial value and potential for securing livelihoods.

## Approaches for data collection

**Fig. 1:**
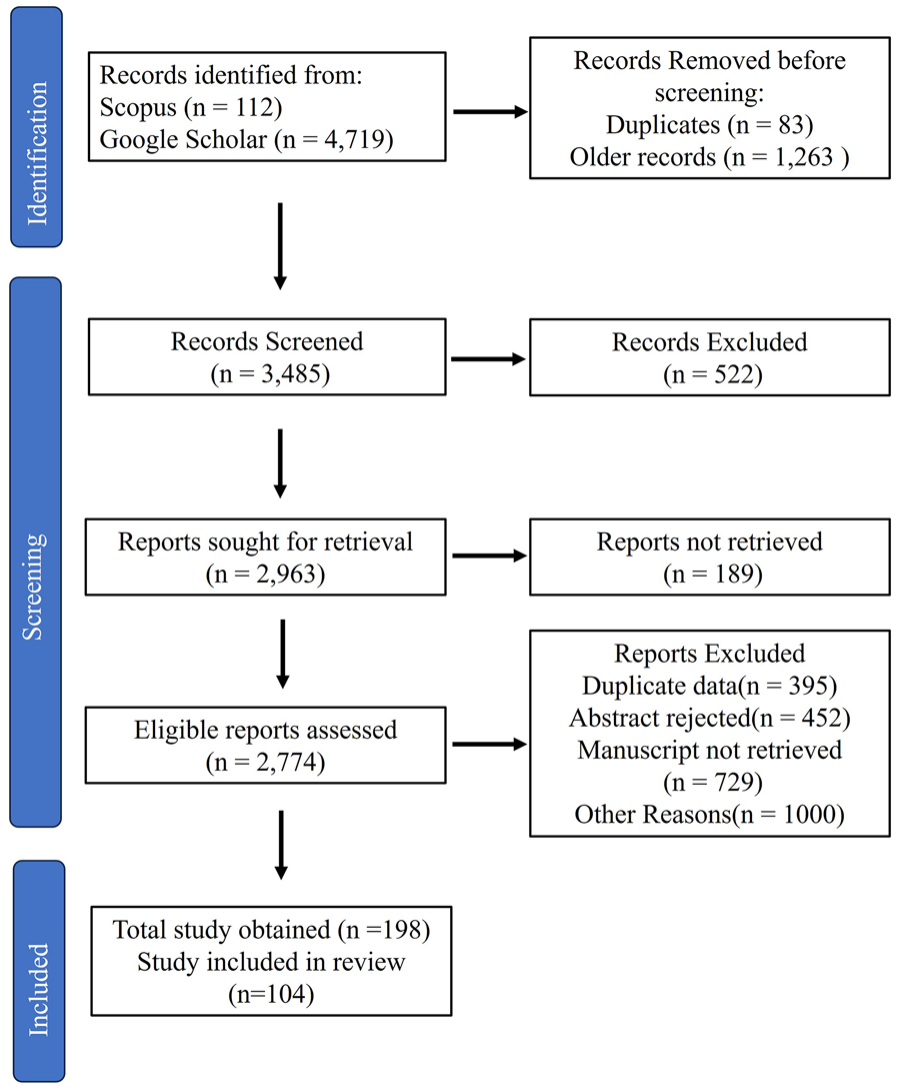
Approaches for data collection for the current review

For the collection of data, a systematic review was conducted using the Scopus and Google Scholar databases. The initial search was based on using various keywords which resulted in a total of 2,690 papers for "*Rubus*" and "Nutraceutical" and "Pharmaceutical", 38 papers for "*Rubus*" and "Pharmacological application" and "Bioactive", 174 papers for "*Rubu*s" and "Nutraceutical" and "Livelihood", 1,680 for "*Rubus*" and "Non-conventional" and 311 for “*Rubus*” and “Superfood” using google scholar database. Similarly, for the Scopus database using the keywords "*Rubus*" and "Non-conventional" resulted in 5 papers, "*Rubus*" and "Pharmacological application" and "Bioactive" resulted in 1 paper, "*Rubus*" and "nutraceutical" and "livelihood" resulted in 1 paper, "*Rubus*", and "Nutraceutical" resulted in 106 papers. The further screening process is given in Fig. 1.

## Traditional applications

Various parts of the plant have been used in traditional medicine to treat different ailments such as digestive problems, skin irritations, diabetes, and fever. *Rubus* species has been used for a long time due to its medicinal properties. Blackberry stems and leaves are used traditionally for wound healing properties, while *R. chingii* fruits are used to improve kidney and liver health [3]. The fruit's antibacterial and anti-inflammatory properties make it a popular choice for natural remedies. The roots of the plant are used in the treatment of jaundice [12]. *Rubus* species are considered to have exceptional nutritional value due to the presence of various bioactive compounds such as tannins, phenols, organic acids, and polyphenols [1, 13] that are useful in various health-promoting-effects, the bioactive constituents from the bark of *Rubus* species used in cosmetic products [3] due to their sun protection and antioxidant activities [14].

## The industrial importance of the *Rubus* genus

### Food and beverages

The fruit is usually consumed fresh, but can also be used to make jams, jellies, fruit juices, toppings, and other confectionery products [15]. The high concentration of secondary metabolites such as phenolics, flavonoids, and terpenoids in the *Rubus* species is what contributes to their classification as "superfoods". Its’ high sugar and carbohydrate contents make it an ideal choice in beverages. In contrast to the most recent study by Liu et al. [16] to make wine using immobilized yeast and free yeast by using red raspberry as substrate. They compared the analysis of both wines based on various properties such as anthocyanin, reducing sugar, and polyphenols. Based on their results the wine made with immobilized yeast resulted in 15% yield and showed better results with total acid 1.6438%, anthocyanins 111.604 mg L^−1^, and polyphenol 565.67 mg L^−1^. Furthermore, their study shows that the sugar consumption rate of immobilized yeast is better than that of free yeast. The fruits belonging to this genus are loaded with various nutritional benefits (Table 1), which are highly perishable and are prone to spoilage, therefore need to be processed for the production of value-added preserved products to take advantage of their nutritional properties.

**Table 1:** Nutritional information of some *Rubus* species (per 100 g) dry weight

Species	Fruit color	Carbohydrate (g)	Proteins (g)	Fiber (g)	Calorie (Kcal.)	References
*R. ellipticus*	Yellow	86.4	4.37	3.53	374.0	[17–19]
*R. ulmifolius*	Red–black	83.62	6.56	1.66	403.29	[17]
*R. niveus*	Black	85.35	3.28	5.90	364.42	[17]
*R. fruticosus*	Black	9.61	1.39	5.3	43	[20]
*R. paniculatus*	Red, Purple	74.29	8.77	–	373.28	[21]
*Rubus fraxinifolius*	Red	11.48	–	6.43	45.92	[22]
*R. rosifolius*	Red	9.86	–	7.10	39.44	[22]
*Rubus pyrifolius*	Dark red	6.84	–	3.36	27.54	[22]
*Rubus chrysophyllus*	Yellow	11.15	–	8.43	44.62	[22]
*Rubus lineatus*	Orange	9.50	–	6.39	38.00	[22]
*Rubus idaeus*	Red	11.94	–	6.50	52.00	[22]

**Fig. 2:**
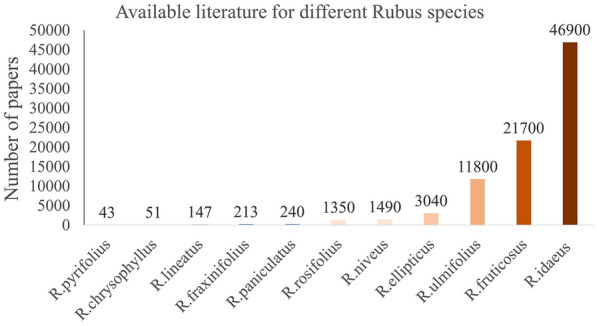
Graphical representation of available literature of different species Rubus

Figure 2 shows the online available literature on Google Scholar (using the keyword “*Rubus* species_name”) which can be used to get information about the work being done on different species. It shows that the most explored species among them is *R. idaeus* (red raspberry). Furthermore, it can be helpful to identify the wild species that need attention to prevent these species from being extinct, until the value addition/similar work as red raspberry of these species is not conducted, people will not understand their benefits.

### Bioactive components in Rubus species

*Rubus* genus exhibits a wide range of secondary metabolites such as phenols, flavonoids, and polyphenols, which depending on the species can vary significantly. The variation in concentrations of bioactive components usually depends upon different factors such as location, cultivar, temperature, etc. [23]*.* This variation can also be observed among different cultivars of the same species and may also differ depending on the location where the plant is grown. For example, According to a study conducted by Bobinait et al. [24] to find the variations in phytochemicals in 19 raspberry cultivars (Black raspberry ‘Bristo’, ‘Putnica’, yellow raspberry ‘Beglianka’ ‘Meeker’, ‘Norna’, ‘Nov-okitaevskaja’, ‘Benefis’, ‘Aborigen’, ‘Glen Moy’, ‘Mirazh’, ‘Lashka’, ‘Siveli’, ‘Polesie’, ‘Husar’, ‘Polana’,‘Polka’, ‘Zorinka’, ‘Ottawa’ and ‘Pokusa’,) from Lithuania. The result of the study showed that the amount of ellagic acid ranged from 119.8 mg 100 g^−1^ (Pokusa) to 323.5 mg 100 g^−1^ (Bristol), while total phenolics varied from 278.6 g 100 g^−1^ (Pokusa) to 714.7 mg 100 g^−1^ (in Bristol). The range of total anthocyanins was from 2.1 mg 100 g^−1^ (Begliianka) to 325.5 mg 100 g^−1^ (Bristol). On the other hand, blackberries were found to contain phenolics 241.7 mg 100 g^−1^ of fruit especially anthocyanins 90.5 mg 100 g^−1^ of fruit and carotenoids 84.5 mg 100 g^−1^ of fruit [25]. Similarly, (50.16 mg Gallic Acid Equivalents(GAE) g^−1^ dw) polyphenolics, (7.73 mg Quercetin equivalent (Qc) g^−1^ dw) flavonoids, and (13.40 mg Cyanidin equivalents (Cy) g^−1^) anthocyanins were reported in the pomace of *Rubus fruticosus* [26]*.* Various analytical techniques are used by many researchers to study the plant chemistry. Mertz et al. [27] used high-performance liquid chromatography with ionization mass spectrometric detection (ESI–MS) and diode array (DAD) in *Rubus glaucus Benth.* and *Rubus adenotrichus Schlech.* from South America and identified many ellagitannins, anthocyanins, and ellagic acids. Similarly, Mertz et al. [28] identified anthocyanins, catechins and ellagic acid derivatives, flavonols, and other compounds from red raspberry and blackberry using HPLC methods( ProStar 330 photodiode array detector, and ProStar 230 solvent delivery module). Similar to these studies many available studies show the presence of various bioactive compounds (Table 2) mostly involving chromatographic analysis [29–31] within the genus.

**Table 2:** Classification of bioactive compounds in various *Rubus* species

Species	Phytocompound	Source	References
Polyphenols			
*Rubus chingii* Hu, *Rubus ellipticus,* raspberry and blackberry	Ellagic acid, caffeic acid, gallic acid, chlorogenic acid, m-coumaric acid,3-hydroxybenzoic acid, 4-hydroxybenzoic acid, vanillic acid, trans-cinnamic acid, kaemferol, cyanin, delphinidin, ( +)-catechin, apigenin, (-)epigallocatechin gallate, galangin, naringenin, quercetin, rutin, sinapaldehyde, hesperidin, pinobanksin, syringaldehyde, tiliroside, astragalin, kaempferol-3-o-β-d-glucuronic acid methyl ester, querecetin-3-o-β-d-glucopyranoside, aromadedrin, hyperoside, cis-tiliroside, kaempferol-3-o-hexoside, quercetin-3-o-glycoronide, imperatorin, rubusin a&b, esculetin, esculin, casuariin, casuarinin, casuaricitin, pedunculagin, caffeic acid glucoside, caffeic acid glucoside, cyanidin 3-o-sophoroside, cyanidin 3-o-glucosyl-rutinoside, cyanidin 3-o-glucoside, cyanidin 3-o-rutinoside, procyanidin dimmer type b, ( +) catechin, ellagic acid rhamnoside, lambertianin c, ellagic acid pentoside, quercetin 3-o-rhamnoside, β-sitosterol	Fruit	[18, 32–35]
*R. ellipticus*	Pyrogallol	Leaves	[18]
*R. ellipticus*	Pinfaensin, rosamutin	Root	[18]
*Rubus fruticosus, Rubus idaeus*	Gallic acid, sanguiin h-6, sanguiin h-10, sanguiin h-2, ellagic acid, ellagic acid pentoside, methyl ellagic acid pentoside	Pomace	[15, 36]
Alkaloids			
*Rubus chingii* Hu, *Rubus ellipticus*	4-Dimethylamino-2,2,6,6-tetramethylpiperidine, 3-piperidinecarboxamide, carbamic acid, n’[3-(dimethylaminopropylamino)propyl]-2-hydroxyimino-2-phenyl, 1,3-propandiamine, 4, 1,1-(diethylcarbamoyl)succinimide, 3-piperidinamine, acetamide, n-[3-(3-dimethylaminopropylamino)propyl]-2-hydroxyimino-2-phenyl, nʹ-[3-(dimethylamino) propyl]-nn dimethyl, 1-(diethylamino)ethylidenimino]sulfur pentafluoride, rubusine, methyldioxindole-3-acetate, 2-oxo-1,2-dihydroquinoline-4-carboxylic acid, 1-oxo-1,2-dihydroquinoline-4-carboxylic acid, 4-hydroxy-2-oxo-1,2,3,4-terahydroquinoline-4-carboxylic acid	Fruits	[18, 34]
Terpenoids			
*Rubus chingi* Hu	Arjunic acid, oleanic acid, fupenzic acid, maslinic acid, sericic acid, ursolic acid, eusaphic acid, 2α-hydroxyursolic acid, 2 α,3 α,19 α-trihydroxyolean-12-ene-28-oic-acid, 2 α,19α-dihydroxy-3-oxo-12-ursen-28-oic acid, tormentic acid, nigaichigoside f1, rubusoside, goshonoside-(F1-F7), ent-labda-8(17),13e-diene-3β,15,18-trio, 27 15,18-di-o-β-d-glucopyranosyl-13(e)-ent-labda-7(8),13(14)- diene-3β,15,18–triol, 15-o-β-d-apiofuranosyl-(1 → 2)β-d-glucopranosyl-18-o-β-dglucopyranosyl-13(e)-ent-labda-8(9),13(14)-diene-3β,15,18-triol	Fruits	[34]
*R. ellipticus*	Ursane, ursolic acid, oleanane	Leaves	[34]
*R. ellipticus*	2R,3,23-trihydroxyurs-12,19-dien-28-oic acid, 2R,3,23-trihydroxyurs-12,18-dien-28-oic acid, buergericic acid, euscaphic acid, r,2r,3,19r-tetrahydroxyurs-12-en-28-oic acid, 19r-hydroxyasiatic acid, 2r,3,19r-trihydroxyurs-12-en-23,28-dioic	Root	[18]
Fatty acids and esters			
*Rubus chingii* Hu, *Rubus ellipticus*	n-Hexadecanoic acid, tridecyl ester, benzenepropanoic acid, 3,5-bis(1,1-dimethylethyl)-4-hydroxmethyl ester, 2-bromopropionic acid, tridecyl ester, hydroxy-, ethyl ester, 1-octacosanol, (E)-9,11-dodecadien-1-ol, [2-(4-hydroxy-phenyl)-ethyl]-carbamic acid ethyl ester	Fruits	[18, 34]
*R. fruticosus L.,R. idaeus*	n-Decanoic acid, dodecanoic acid, hexadecenoic acid, linoleic acid methyl ester	Leaves	[18, 34]
Phytosterols			
*Rubus fruticosus, Rubus idaeus*	Stigmasterol, campesterol, β-sitosterol	Pomace	[15, 36]
*R. ellipticus*	β-Sitosterol, β-sitosterol- β-D-glucoside	Leaves	[18, 34]
Other compounds			
*Rubus chingii* Hu, *Rubus ellipticus*	Phloridzin, 4-methylumbelli-ferone, p-aminobenzoic acid, 7,9-dimethyl-1,4-dioxa-7,9-diazacycloundecan-8-one, 4(equat)-n-butyl-1,2(axial)-dimethyl-transdecahydroquinol-4-ol, ascorbic acid (vitamin c), glurolactone, vitamin e	Fruit	[18, 34]
*Rubus chingii* Hu, Rubus ellipticus	α-Terpinene, 2,2,4-trimethyl-pentane, linalyl acetate, 2,2,3,3-tetramethyl-butane, 1-hydroxy-2-methyl-1-phenyl-3-pentanone, α-thujene, trans-linalool oxide, l-α-terpineol, 1-octacosanol, octacosanic acid, 2-ethylhexyl acrylate, cis-linalool oxide 2-(2-butoxyethoxy)-ethanol acetate, diisobutyl phthalate, phytol, 3-methyloctanedioic acid-dimethyl ester, dodecyl aldehyde, 1-(4,7,7-trimethyl-3-bicyclo[4.1.0]hept-4-enyl)ethenone, e-10-pentadecenol, trans-dihydrocarvyl acetate, coniferyl alcohol, 1-(4-hydroxymethylphenyl)ethenone, terpineol-4, cis-p-2-menthen-1-ol, (e)-1-(2,6,6-trimethyl-1,3-cyclohexadien-1-yl)-2-buten-1-one, calarene, cedryl formate, 12-methyltridecanal, 5-oxoheptanoate methyl, neryl acetate, n-tridecane, trans-caryophyl-lene, quinic acid, 2-hexenal, 2,4-heptadienal, 2-hydroxy-5-methylbenzaldehyde, 5-(hydroxymethyl)furfural, 2-heptanone, 2-hexanone, 2-hexanol-3-methyl, 4-heptanol-3-ethyl, 3-hexanol-5-methyl	Leaves	[18, 34]

Ellagic acid, gallic acid, chlorogenic acid, ferulic acid, m-coumaric acid, 4-hydroxybenzoic acid, 3-hydroxybenzoic acid, vanillic acid, trans-cinnamic acid, phloridzin, caffeic acid, cyanin, delphinidin, β-carotene, ascorbic acid, 4-dimethylamino-2,2,6,6-tetramethyl piperidine are some the common compounds (Table 3) that are found in *Rubus* species fruits [37, 38]. The presence of amino acids such as L-hydroxyproline, DL-2aminobutriyic acid, DL valine, DL Iso-leucine, L-Cysteine hydroxyl, DL alanine, DL-nor-leucine, L-glutamic acid, DL-aspartic acid, L-arginine, DL-aspartic acid, L-cysteinhydroxychloride, L leucine, DL-methionine, L-Lysine, DL-threonine, L-tyrosine have also been reported in *Rubus ellipticus* [10].

**Table 3:** Commonly found phytocompounds of *Rubus* species and their applications

Compounds	Structure	Biological activity	Food industry	Other applications	References
3-Hydroxybenzoic acid		Derivatives of this compound have antimicrobial, antimutagenic antiestrogenic, hypoglycemic anti-platelet aggregating, antiviral, anti-inflammatory	–	Plasticizers, resins	[39]
Ascorbic acid		Antioxidant, anticancer, Wound healing	Preservative, anti-browning agent, Prevents oxidation and discoloration of the meat	Cosmetics	[40–42]
Caffeic acid		Anti-cancerous, Hepatocarcinoma, Anti-oxidant, anti-inflammatory	Meat preservation	–	[43–46]
Chlorogenic acid		Obesity, anti-diabetic, Dyslipidemia, hypertension, Anti-bacterial	Whey protein conjugation, Food additives, extended food storage life, food packaging, prebiotics,	Cosmetics	[46–48]
Delphinidin		Anti-cancerous, neuroprotective, cardioprotective, antidiabetic, anti-hepatotoxic, antiviral	–	The active ingredient in Hair loss prevention, and the separation of delphinidin derivatives from eggplant peel	[49, 50]
Ellagic acid		Antioxidant, Antibacterial, antileishmanial, antimalarial, Antifungal, Gastroprotective, antianxiety	–	Chelating reagents, copolymers, ion-exchange resins, conductivity-based sensors	[51]
Ferulic acid		Antioxidant, anti-inflammatory, antimicrobial, antiviral, vasodilatory, antithrombotic, and antiallergic	Food preservative	Skincare and Cosmetics production	[52, 53]
Gallic acid		antiallergic, anti-inflammatory, antimutagenic and anticarcinogenic, antioxidant, antiulcer	Prevents rancidity and spoilage of oils and fats, baked products, candy, and chewing gums	Cosmetics, standard for total phenol content, used in photography, dyes, and printing	[54–56]
m-Coumaric acid		Antioxidant, anti-inflammatory, anti-cancerous	–	–	[56]
Phloridzin		Antihyperglycemic, antioxidant, antioxidant, hepatoprotective, antitumor, antibacterial, cardioprotective	Food additives, food, and beverage preservative	Cosmetics, a biomarker for phloridzin-containing foods	[57]
Trans-cinnamic acid		Anti-cancerous, Antibacterial, antidiabetic, neurological disorders	–	Flavoring cosmetics and detergents	[58]
Ursolic acid		Anti-carcinogenic, anti-apoptotic, anti-inflammatory, antioxidant, antirheumatic, antitumoral, antiviral,	–	Herbicidal,	[59]
Vanillic acid		Sedative, antidepressant, antinociceptive, hypertension, anti-cancerous, antifungal, anti-inflammation, wound healing	Flavoring agent	–	[60–62]
Vitamin E		Antioxidant, anti-platelet coagulation	Dietary supplements, prevent the oxidation of foods and increase shelf-life, fortification of foods,	Cosmetics,	[63]
β-Sitosterol		Antioxidant, anti-diabetic, antimicrobial, anti-inflammatory, anti-HIV, anti-pulmonary, anti-arthritic, antipyretic	–	–	[64]

**Fig. 3:**
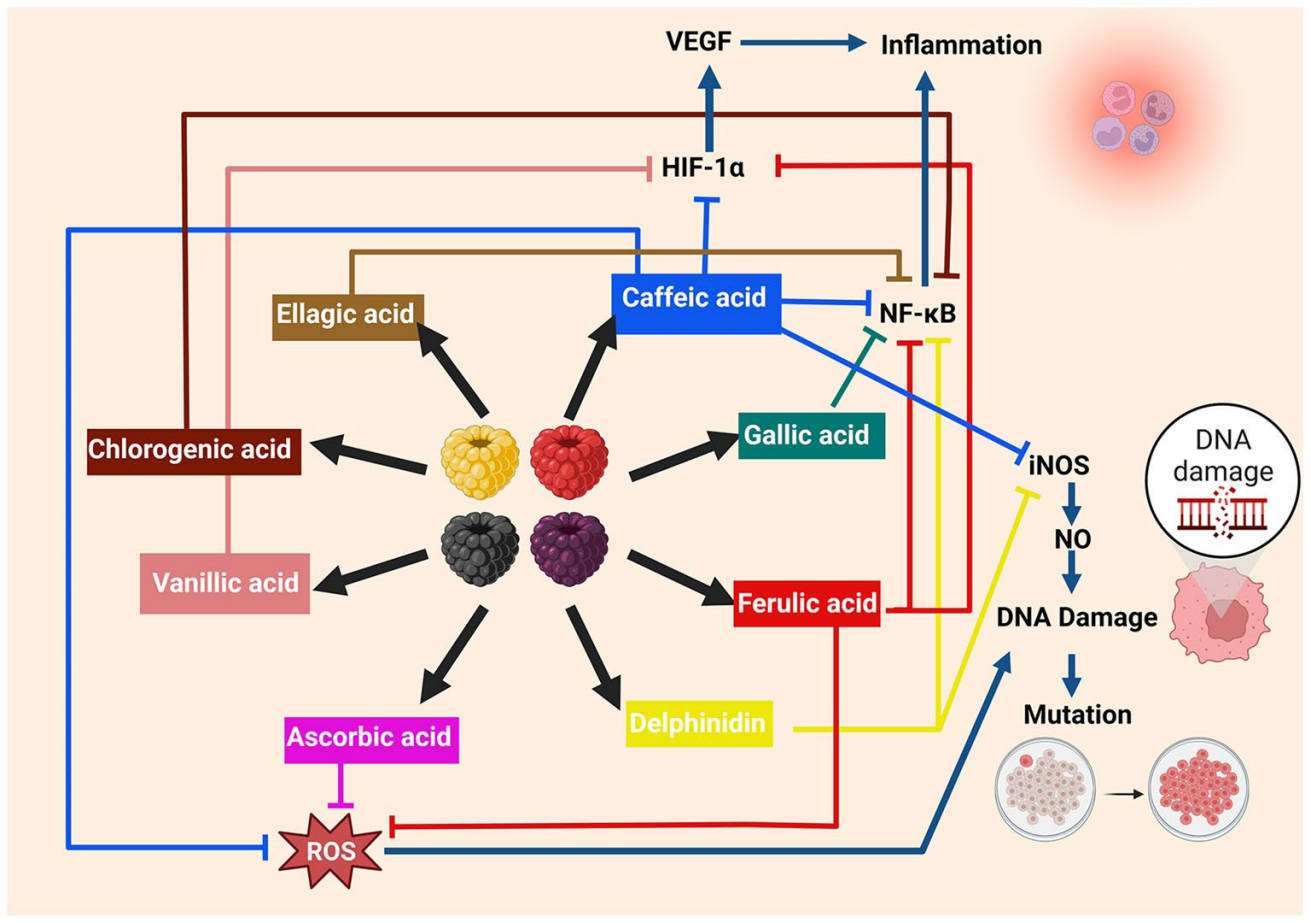
Mechanism of action of various phytocompounds of *Rubus* species The figure depicts a schematic representation of various phytochemicals found in *Rubus* species and their respective mechanism of action; *VEGF* vascular endothelial growth factor, *HIF* hypoxia inducing factor, *ROS* reactive oxygen species, *NO* nitric oxide, *iNOS* inducible nitric oxide synthase

The Fig. 3 depicts a schematic representation of various phytochemicals found in *Rubus* species and their respective mechanism of action. Ferulic acid, gallic acid, caffeic acid, ellagic acid, delphinidin, and chlorogenic acid play a significant role as anti-inflammatory agents by inhibiting the responsible factor NF-κB. Similarly, caffeic acid, ferulic acid, and vanillic acid show anti-inflammatory activity by inhibiting the HIF-1α while the role of ascorbic acid as an antioxidant, and caffeic acid, as an anticancer agent works by inhibiting the ROS generation and preventing DNA damage and mutation[34–59].

### Medicinal importance

The medicinal potential of the *Rubus* genus has been explored by many researchers and it is not only limited to its fruits. Phytochemical analysis of several *Rubus* species has revealed the presence of various compounds such as polyphenols, flavonoids, and anthocyanins. Previous research has shown that this species contains a compound with antioxidant properties, leading to overall health benefits. It has been reported to have anti-inflammatory, analgesic, antipyretic, antidiabetic, anti-tumor, wound-healing, anti-cancer, and antibacterial effects [1, 65, 66]. The plant has been used for many generations in the treatment of various ailments such as gastrointestinal illness, diabetes, bacterial and fungal infections, wound healing, and ulcers [1].

#### Antidiabetic activity

Boscaro et al. [67] have mentioned that pre-diabetic women with insulin resistance and obese, metabolically healthy individuals who consumed red raspberries, and *Rubus ellipticus* ethanolic extracts of fruits respectively exhibited a reduction in peak glucose and insulin levels, as well as lower 2-h glucose area under the curve and improved glucose tolerance in rats [68]. This antihyperglycemic effect is achieved through various mechanisms, including β-cell regeneration, glucose-load reduction, increased insulin action and secretion, and receptor activation, resulting in improved blood sugar uptake and peripheral glucose consumption [69]. Furthermore, the extract from *Rubus* fruit has been found to exhibit hypoglycemic effects and enhance insulin sensitivity in Sprague Dawley rats, while also promoting lipid catabolism in adipose tissue, with a more pronounced effect observed in female rats [70]. Additionally, the gut metabolites of blackberries have been found to increase the consumption of glucose and glycogen content and improve high glucose plus palmitic acid-induced ROS overproduction, mitochondrial membrane damage, and glutathione depletion in HepG2 cells [71]. The intake of *R. idaeus L.* fruit has also been reported to have antidiabetic effects and is effective against oxidative stress associated with type 2 diabetes in mice by enhancing glutathione peroxidase(GPx)/superoxide dismutase(SOD) ratio and preventing oxidative stress [72]. Safarzad et al. [73] investigated the effects of a leaf extract from *R. anatolicus* in CRI-D1 cell line and conveyed that the extract helps to improve the consumption and uptake of glucose while enhancing the secretion of insulin in pancreatic cells. The finding of Ayele et al. [74] shows that the leaf extract of *Rubus erlangeri* exhibits significant effects on lowering the blood glucose level without weight loss and hypoglycemia.

#### Antioxidant activity

Several studies have investigated the antioxidant and antimicrobial activities of *Rubus* plants. Matawali & Azlan [75] reported that the leaves of the plant exhibited the highest antioxidant capacity compared to other plant parts, with the methanol extract showing the highest antioxidant activity. According to their study, the total phenolic content (56.32 ± 0.05 mg/ (GAE)/g) and total flavonoid content (31 ± 1.05 mg Catechin equivalents (CE)/g) were found to be higher in the leaves while the total anthocyanins content (22.27 ± 1.28 × 10^–14^ mg cyanidin-3-glucoside equivalent (C-3-GE) /g) and total carotenoid content (10.02 ± 0.22 mg β-carotene equivalents (BC)/g) was found higher in fruits. The presence of various secondary metabolites is responsible for various types of pharmacological activities, in contrast, the radical scavenging activity of *Rubus* species(Raspberry, and blackberry) leaves using DPPH assay was found 105.2 mg Ascorbic Acid Equivalents (AAE)/g for raspberry while 152 mg AAE/g for blackberry leaves [76].

#### Antibacterial activity

Saini et al. [77] reported that fruits of *R. ellipticus* did not show any antibacterial effects against *Staphylococcus aureus, Bacillus subtilis,* and *Escherichia coli*. On the contrary Velićanski et al. [78] found that fruits of *Rubus idaeus* exhibited antibacterial activity against *Pseudomonas aeruginosa* and *Bacillus cereus*. Kumar et al. [79] observed that the extract of *R. ellipticus* fruit in methanol, hydro-alcohol, and hexane had significant antimicrobial activity against *Micrococcus luteus, Escherichia coli, Salmonella abony, Staphylococcus epidermidis, Aspergillus niger,* and *Candia albicans*.

#### Anti-cancerous activity

Velićanski et al. [77] have demonstrated the ability of *R.ellipticus fruit* extract to inhibit cell proliferation in two cervical cancer cell lines (C33A and HeLa) and a normal cell line Peripheral Blood Mononuclear Cells (PBMCs) using MTT assays. The results showed that the extract had strong antiproliferative effects on C33 cells in a dose-dependent manner, while no inhibitory effect was observed against HeLa cells. Additionally, the extracts did not exhibit any cytotoxic effects on PBMCs and instead promoted their growth. These findings indicate that *R.ellipticus* fruits have selective anticancer properties against cervical cancer cells while being non-toxic to normal cells. The methanol and acetone extracts were more effective than acidic extracts, with lower EC_50_ values against C33A cells [77]. Anticancer properties of *R.chingi* was observed by Li et al. [80], their results showed that *R.chingi* showed potent antioxidant properties and could inhibit the growth of T24 bladder cancer cell in a dose-dependent manner by inducing apoptosis. It has been reported that the extract obtained from *Rubus* fruits can inhibit the proliferation of several human tumor cell lines, including oral (KB, CAL-27), colon (HT-29, HCT116), breast (MCF-7), and prostate (LNCaP) cells. The study also demonstrated the efficacy of the extract in inducing apoptosis in a colon cancer cell line expressing COX-2 [81].

## Livelihood security

Red and black raspberries, blueberries, and blackberries are the most economically important *Rubus* species grown for the fresh market and processing. In 2017, the global production of red and black raspberries was 840,000 tonnes, with Europe and the Americas being the top producers [2]. In the year 2020, the production of raspberries was found to dominate over strawberries and blackberries [82]. In 2020 the topmost imported fruits from Mexico were avocados and berries including blueberries strawberries and raspberries. These fruits collectively made up around 1/3 (32%) of the total value of imported fruits from Mexico. Among these fruits blueberries show the most significant growth in imports, increasing from 6 million USD to 352 million USD during the same period. It shows the increasing dependency of people on berries. Non-conventional fruits can be a good source of alternative income. Hence *Rubus* species can also provide livelihood opportunities for many people as they can be cultivated commercially or collected from the wild. This may provide a source of income for farmers, small-scale growers, and forgers. In India, non-conventional fruits (wild fruits) have a significant impact in terms of fulfilling the food and cash income needs of rural, poor, and tribal populations [83]. Similarly, in Ecuador, the cultivation of *R. glaucus* has also been shown to contribute to the livelihood of a substantial number of medium and small-scale producers, with an estimated 12,000 individuals benefiting from this [84]. Jalisco, Mexico is the main producer of raspberry and blackberry at the national level. Hence the wild species of *Rubus* also needs to be explored for its nutritional and nutraceutical properties. The preservation of germplasm and promotion of conventional cultivation for wild species of *Rubus* can provide economic advantages, as well as nutritional and livelihood security, further, the use of *Rubus* species can be explored for industrial purposes and value addition.

## Value addition and industrial aspects

*Rubus* species has a long history of use for both food and medicinal purposes. Chutney made from blackberry was found to have reduced anthocyanins, phenolics, and antioxidants compared to the pulp but were still well-accepted in sensory analysis [85]. A study by Felix da Silva et al. [86] investigated changes in phenolic compounds and antioxidant activity during the storage of jam, coulis, and purees made from Brazilian cultivar blackberries. The results showed a decrease in total phenolic content in jam but an increase in antioxidant activity, suggesting the potential for chemical transformation of compounds with higher antioxidant activity. Similarly, Souza et al. [87] combined black, red, and yellow raspberries to make jellies and found that the sensory acceptability of these jellies was higher than jellies made from 100% black or yellow berries. Berries are also good sources of carbohydrates, making them suitable for use in alcoholic beverage production. Red raspberry and arbutus berries have been used as substrates in fermentation for producing safe, high-quality beverages without adverse health effects from methanol concentrations [88]

Industrial processing of berries into value-added products generates another by-product (which is mostly seeds) called pomace. Typically, this by-product is considered waste although it contains crucial bioactive compounds and unsaturated fatty acids such as (PUFAs) [15] and can be transformed into value-added products such as functional and nutraceutical food products [36]. It was reported that raspberry pomace contains around 77.5% of the total dietary fiber of total fruit [89]. Some researchers have mentioned creating additional products from the berry pomace. Tarasevičienė et al. [90] and Peiretti et al. [91] have made patties using berry pomace. Both studies concluded that the pomace helps to obtain desirable changes in the product and acts as a thickener while delaying the oxidation of lipids.

## Recent developments and future outcomes

There are several ongoing research in *Rubus* species. Some of the developments related to the *Rubus* genus including advances in breeding [92], making value-added products [93], preservation methods [94], and synthesis of nanoparticles [95, 96] are summarized in Table 4. The future outcome for this genus involves Post-harvest management methods to avoid waste during the processing of *Rubus* into by-products as the fruits are delicate and prone to damage. Additionally, there are opportunities to develop by-products using plant parts such as fruits, leaves, and pomace. For example, pomace can be reused as animal feed, biofuel production, and composting. Furthermore, the genetic diversity of this genus needs a deeper understanding. Current breeding and commercialization efforts mostly involve a few well-known species like black raspberry, and red raspberry, exploring other underutilized plants of this genus can pave new pathways that can potentially enrich the food plate while expanding the germplasm archival of this group.

**Table 4:** Recent research and development related to *Rubus species*

Plant species	Methods	Research findings	References
*Rubus idaeus*	Acid, alkaline, and enzymatic hydrolysis to improve the release of phenolic compounds	All methods significantly improve the isolation of phenolics while acid hydrolysis was the most efficient	[97]
*Rubus fruticosus*	Extraction of antioxidants, and microencapsulation with maltodextrin by spray dryer to check stability at different pH	Maltodextrin was found to be efficient in reducing the anthocyanin loss against increased pH, greater stability was observed at lower pH. A reduction in the percentage loss of antioxidants was observed using water for the extraction	[98]
*Rubus humulifolius*	Cryopreservation protocol for critically endangered *R. humulifolius* in Finland	A method of cryopreservation was developed by incorporating abscisic acid resulting in enhancement of cryopreservation for critically endangered *R.humulifolius*	[94]
*Rubus fairholmianus*	Synthesis of zinc oxide nanoparticles using *R. fairholmianus* root extract	The nanoparticles exhibited strong antimicrobial activity against *Staphylococcus aureus*	[95]
*Rubus* species	Genome sequencing	8 new chloroplast genomes were obtained, which might be helpful in phylogenic analysis of the genus	[99]
*Rubus* species	Three methods (spray-drying, freeze-drying, and ionic gelation) were used for microencapsulation of blackberry pomace and evaluated for stability in the absence/presence of light	Spray-dried microcapsules had the highest stability and bioavailability of anthocyanins in yogurt formulation, suggesting the potential for high-value food products	[93]
*Rubus corchorifolius*	Inhibitory activity assay using raspberry leaf-tea extract, molecular docking studies	Inhibition of α-glucosidase and α-amylase, the ethanolic extract improved glucose consumption of 3 T-3L1 cells	[100]
*Rubus idaeus*	Homeopathically prepared *Rubus idaeus* was studied for the treatment of cervical cancer using viability, proliferation, and cytotoxicity, assay with laser treatment in HeLa cells	*Rubus idaeus* extract (D3) able to reduce cellular viability, combined with laser showed an increase in adenosine triphosphate (ATP) and lactate dehydrogenase (LDH) levels	[101]
*Rubus ellipticus*	Actinomycetota isolated from the rhizospheres of two (*R. ellipticus*, *Ageratina riparia*) plants for their antimicrobial and plant growth-promoting activities	Plant-associated actinomycetes show potential as bioinoculants for increased crop productivity and food security	[105]
*Rubus ellipticus*	Synthesis and characterization of Cu_2_O nanoparticles using green nanotechnology	The nanoparticles show good antimicrobial and anticancer properties with low toxicity, indicating the potential in the pharma and food industry	[96]
*Rubus occidentalis* and *Rubus parvifolius*	Development of interspecific hybrids using simple sequence repeat markers and colchicine treatments	Successful development of tetraploid providing a new genetic resource for environmental adaptability	[92]

## Conclusion

The wide range of genetic diversity in *Rubus* species holds significant promise for creating value-added products, making them a compelling alternative to conventional types, such as raspberry, and blackberry*,* in diverse fields. In conclusion, the review of the scientific literature on *Rubus* species highlights the potential of fruit as a source of bioactive compounds with diverse health benefits. The fruit has been reported to possess antidiabetic antioxidant, antibacterial, and anti-cancerous effects, which can be attributed to its polyphenols, anthocyanins, and other phytochemicals. These properties are beneficial for both the pharmaceutical and food industry. Moreover, the fruit can be processed into jams, jellies, and other confectionary food products due to its unique flavor and aroma. The popularity and the market demand of the fruits from cultivated species of *Rubus* genus is increasing day by day due to their nutritional and industrial importance. The fruits of the *Rubus* genus that are more popular, such as red raspberry, and blue raspberry, have increasing demand daily, while the wild, edible, non-conventional fruits are limited to local livelihood. Non-conventional fruits of the same genus are limited to the local livelihood. A better understanding of the wild varieties of this genus, such as *R.ellipticus*, *R.paniculatus*, and *R.ulmifolius,* etc. Although many species of Rubus are globally cultivated and well-explored for their nutraceutical properties and industrial aspects, there are still some commercially unexplored species, including *R.ellipticus, R.niveus, R.paniculatus, R.fraxinifolius, R.pyrifolius, R.chrysophyllus, R.lineatus*, etc. that needs more attention. This is especially important as these fruits are seasonal and are available for a limited period. Although the available research has established the potential benefits of *Rubus* species, still at the industrial level there are only some major species that are being used for their value addition. Since the wild species do not contribute to the economy, people have started to ignore their existence, or only use them within individual communities. If this continues these species will fall into the endangered categories. Further studies are required to explore its full therapeutic and industrial potential.

## Data Availability

Not applicable.
